# Exploring the Link between Hydrodynamic Size and Immunoglobulins of Circulating Immune Complexes in Rheumatoid Arthritis Patients

**DOI:** 10.3390/ijms25063138

**Published:** 2024-03-08

**Authors:** Tamara Djukić, Ivana Drvenica, Marijana Kovačić, Sladjan Milanović, Dragana Majerič, Mirjana Šefik-Bukilica, Maja Miletić, Branko Bugarski, Vesna Ilić

**Affiliations:** 1 Innovation Center of the Faculty of Technology and Metallurgy Ltd., 11000 Belgrade, Serbia; tdjukic@tmf.bg.ac.rs; 2 Institute for Medical Research, University of Belgrade, National Institute of Republic of Serbia, POB 39, 11129 Belgrade, Serbia; marijana.buac@imi.bg.ac.rs (M.K.); sladjan.milanovic@imi.bg.ac.rs (S.M.); vesnai@imi.bg.ac.rs (V.I.); 3 School of Dental Medicine, University of Belgrade, 11000 Belgrade, Serbia; dragana.smiljanic.989@gmail.com (D.M.); maja.miletic@stomf.bg.ac.rs (M.M.); 4 Institute for Rheumatology, 11000 Belgrade, Serbia; sefikbukilicam@yahoo.com; 5 Faculty of Medicine, University of Belgrade, 11000 Belgrade, Serbia; 6 Faculty of Technology and Metallurgy, University of Belgrade,11000 Belgrade, Serbia; branko@tmf.bg.ac.rs

**Keywords:** dynamic light scattering, rheumatoid factor, autoantibodies against citrullinated peptides, circulating immune complexes, immunoglobulin content

## Abstract

The function of immune complexes in rheumatoid arthritis (RA) is related to their composition and size. Using dynamic light scattering (DLS), we investigated the link between the RA circulating immune complex (CIC) particles’ size and the CIC immunoglobulin level. In this study, 30 RA patients and 30 healthy individuals were included. IgA, IgG, and IgM were found in all analyzed CICs, but more IgA and IgG were found in RA than in control CICs. In both control and RA CICs, DLS detected 50 particles that differed in size and clustered around two size groups: with a 7.5–164 nm radius and with a 342–1718 nm radius. An increased level of IgA in RA CICs, compared to control ones, was associated with more than 50% of CIC particles. In RA, compared to the control, a higher number of CICs with 28.2 nm, 531 nm, 712 nm, and 1718 nm particles and a lower number of CICs with 78.8 nm particles were detected. This particle distribution pattern did not reflect the changes in the CIC immunoglobulin level. Thus, RA elevated CIC IgA was linked with all these particles (except the 1718 nm particle), the IgM increase was linked with 43.8 nm and 712 nm particles, and the IgG increase was linked with the 712 nm particle only. This study provides the very first data on the association between CIC particles’ size, CIC immunoglobulin level, and RA. It opens the possibility that the size of CICs determined by DLS can be used as a criterion in RA diagnosis or monitoring after a large-scale study confirmation.

## 1. Introduction

The main characteristic of humoral immune response in rheumatoid arthritis (RA) is the presence of increased levels of rheumatoid factors (RFs), autoantibodies against epitope expressed in the Fc region of IgG molecules upon antigen binding, and autoantibodies against citrullinated peptides (ACPAs) [1,2]. The presence of antibody–antigen complexes, i.e., immune complexes (ICs) containing RFs [3] and ACPAs [4], in the peripheral blood and inflamed joints of RA patients was reported several decades ago [3,4]. Previous studies showed that synovial fluids generally contain a higher level of ICs than paired sera and that the level of ICs in peripheral blood is not necessarily increased, especially in early RA [5,6,7,8]. However, the immunomodulatory functions of ICs are dependent not only on their concentration but also on their size and molecular composition. It has been shown that even minor changes in the structure of ICs significantly affect their functional capacity [9], which points out the necessity of the study of IC arrangement and organization even if their concentration is not significantly increased. The importance of immunoglobulin isotypes as structural elements of ICs in RA has been confirmed by studies showing that IgG-containing ICs regulate bone homeostasis [10] and especially IgA-containing ICs induce osteoclast-mediated bone resorption through IgA-FcαRI interaction [11]. IgA-containing ICs are also potent activators of neutrophils and inducers of neutrophil extracellular traps (NET) formation [12] and local inflammation in RA. 

The investigation of the physical parameters of ICs, such as the size of ICs in RA, needs to be improved, despite the initial studies acknowledging the potential of measuring the size of circulating ICs as a diagnostic tool to assess disease activity [3,5,13]. György and co-workers [14] demonstrated the practicability of dynamic light scattering (DLS) for measurements of IC size in the synovial fluid of RA patients. They showed that IgG- and IgM-containing ICs, isolated by affinitive chromatography from pooled synovial fluid of RA patients, differed in particle size. Inspired by the work of György and others [14], we have recently demonstrated the feasibility of DLS to identify the RA-specific but also the age-specific distribution of pooled circulating immune complex (CIC) size using sera of RA patients [15]. Additionally encouraged by the fact that data on the association of RA disease profiles with immunoglobulin classes/subclasses/allotypes are also contradictory [16,17], in this study, we were motivated to determine the size of colloid particles in the CICs of individual RA patients by DLS and to analyze if the presence of ACPAs and RFs in peripheral blood, and the specific immunoglobulin isotypes in isolated CICs, were associated with particular sizes of CIC colloidal particles. Exploring the link between different classes of immunoglobulins and the sizes of CIC particles in RA could deepen our understanding of the disease’s immunopathology, potentially contributing to the development of improved diagnostic methods and targeted therapeutic interventions for RA.

## 2. Results

In this study, we have analyzed CICs of 30 RA patients and 30 healthy middle-aged and older adults, representing RA age-matched control. The data on RA patients’ age, gender, RF and ACPA levels, erythrocyte sedimentation rate (SER), C reactive protein (CRP) level, and disease activity (DAS28 score), as well as the control group’s age and gender, are given in Table 1 and Table S1 (Supplementary Materials). Based on ACPA and RF status (Table S1, Supplementary Materials), the patients were classified into two main groups: ACPA+ RF+ (the patients with concomitantly positive RF and ACPA; twenty patients) and ACPA-RF- (ACPA-RF-: having no detectable level of RF and ACPA; six patients). In addition, three patients were ACPA+RF- and one was ACPA-RF+. A significant correlation between ACPA and RF titers was observed in a group of RA patients. Within the same cohort, no significant correlations were discerned between the titers of these autoantibodies and indicators of inflammation, such as SER and CRP levels, nor with measures of disease activity (DAS28 score) (Table S2, Supplementary Materials). None of the RA patients were treated with therapeutic monoclonal antibodies, and the disease-specific therapy received at the time of the blood collection is given in Table S1, Supplementary Materials. Although RA can impact various organs, including the lungs and cardiovascular system, either through direct autoimmune pathology or chronic inflammation [18], clinical data from this study indicate an absence of extra-articular manifestations in the RA patient cohort examined. The control group participants had no documented systemic diseases or acute infections one month before the blood sampling.

### 2.1. CIC Immunoglobulin in RA

**Figure 1:**
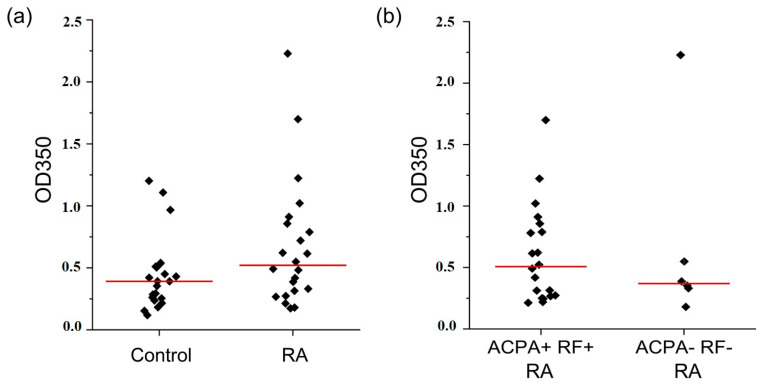
The level of CIC in (**a**) RA patients and RA age-matched control group of healthy people and (**b**) the level of CIC in ACPA+RF+ and ACPA-RF- RA patients. Red lines: median values.

The level of CIC isolated by PEG precipitation in RA patients was 0.593 ± 0.457 (median value: 0.407) and did not significantly differ from the level of the control group (average value: 0.464 ± 0.307; median value: 0.450) (Figure 1a). The presence of RF and ACPA autoantibodies in sera did not impact the CIC level in RA patients (Figure 1b). The individual CIC level (expressed as OD350 values) is given in Table S1, Supplementary Materials. No significant correlation was found between the CIC level and DAS28 values (Supplementary Materials, Table S3). Additionally, no significant impact of different RA treatments (methotrexate and prednisone vs. methotrexate, prednisone and disease-modifying antirheumatic drugs (DMARDs) vs. methotrexate and DMARDs) on the CIC level was detected (*p* = 0.73).

**Figure 2:**
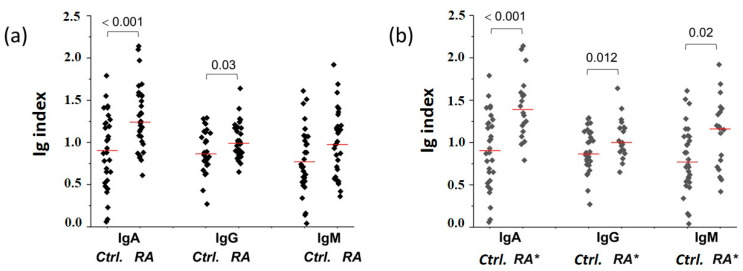
Relative content of immunoglobulins in CICs of RA patients. (**a**) Relative content of IgA, IgG, and IgM in CICs of RA patients (RA) and in CICs of RA age-matched control group of healthy people (Ctrl.). (**b**) Relative immunoglobulin content in the control CICs (Ctrl.) and CICs of ACPA+RF+ RA patients (RA*). Ig index: relative immunoglobulin content, determined by dot blot. It represents the intensity of CIC IgG, IgA, or IgM dot blot signals normalized to the assay control (pooled healthy human serum). Red lines: median values.

Using a dot blot assay, we confirmed the presence of IgA, IgG, and IgM in all analyzed CIC samples and found that the relative content of IgA and IgG, but not IgM, was higher in the CICs of RA patients than in healthy control CICs (Figure 2a). The CICs of ACPA+RF+ RA patients contained, compared to the control, significantly higher levels of all immunoglobulin classes (Figure 2b). The individual values for CIC IgA, IgG, and IgM are given in Table S1, Supplementary Materials.

To evaluate the strength and direction of the correlation between obtained Ig indexes and the level of CICs, we performed a correlation analysis (Table S3, Supplementary Materials). In the control group, the CIC level positively correlated with the relative content of all immunoglobulin classes. However, in the RA group, the CIC level positively correlated with IgM (r = 0.65, *p*< 0.001; Table S3, Supplementary Materials).

The level of CICs did not show any correlation with either ACPA or RF titer in the RA group (Table S3, Supplementary Materials). While the serum RF titer correlated with the level of all classes of CIC immunoglobulins (IgA r = 0.51, *p* = 0.004; IgG r = 0.37, *p* = 0.046; IgM r = 0.38, *p* = 0.036; Table S3, Supplementary Materials), the serum ACPA titer correlated only with the level of CIC IgA in the RA group (r = 0.45, *p* = 0.012; Table S3, Supplementary Materials). The ACPA+RF+ RA group of patients revealed a negative correlation between the level of CICs and the titer of serum ACPAs (r = −0.62, *p* = 0.003; Table S3, Supplementary Materials) and a positive correlation between the titer of serum RFs and the CIC IgG level (r = 0.46, *p* = 0.04; Table S3, Supplementary Materials).

**Figure 3:**
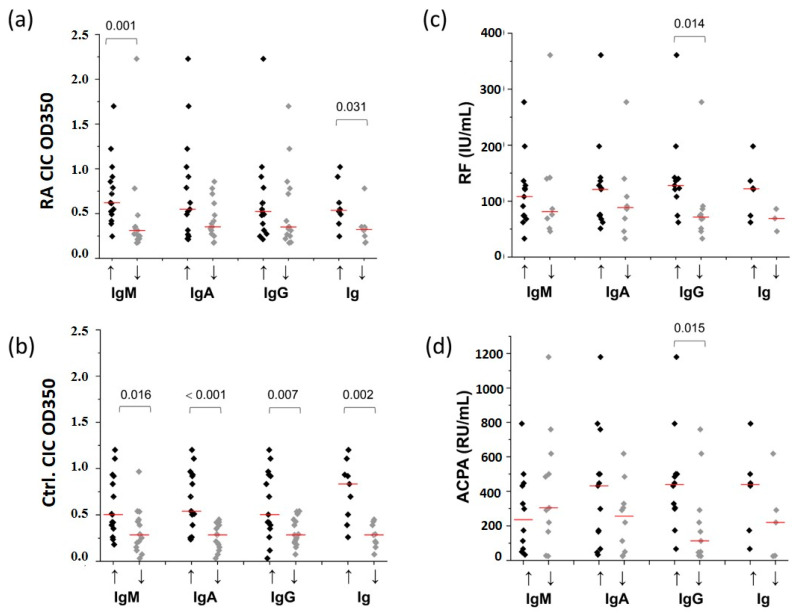
The link between the CICs’ immunoglobulin content and the CIC level in RA patients (**a**) and healthy people (**b**) and between the CICs’ immunoglobulin content and the serum titer of RF (**c**) and ACPA (**d**) in RA patients. IgA↑, IgM ↑, IgG ↑—the CIC samples with the IgM, IgG, or IgG level above the median value (black rhombuses); IgA ↓, IgM ↓, IgG ↓—the CIC samples with the IgM, IgG, or IgG level below the median value (grey rhombuses); Ig ↑ and Ig ↓—CIC samples having all immunoglobulin classes above (black rhombuses) or below (grey rhombuses) the median value. Red lines: median values.

In accordance with the results of the correlation analysis, we have shown a significant link between the level of CICs and the relative content of all immunoglobulin classes, as well as the total immunoglobulins in the control group (Figure 3b). On the other hand, a significant association was only found between the level of CICs and the relative content of CIC IgM and total CIC immunoglobulins in RA patients (Figure 3a). In addition, a higher level of CIC IgG, but not of CIC IgA and CIC IgM, was associated with higher serum ACPA (Figure 3d) and RF titer (Figure 3c). The presence or absence of ACPAs and/or RFs in the serum of RA patients was not associated with an increased level of immunoglobulins in their CICs (Table S4, Supplementary Materials).

### 2.2. DLS Analysis of Colloidal Particles’ Size in CICs in RA

In our previous research, we successfully applied DLS to determine CIC particles’ size in pooled CIC samples of healthy people and RA patients [15]. Herein, we performed DLS measurements on individual CIC samples for unbiased and highly accurate analysis.

First, we mapped the sizes (i.e., hydrodynamic radii) of colloidal particles in the CICs of RA patients and RA patients’ age-matched control group. In both groups, 30 individual CIC samples were analyzed by ten repeated particle size measurements, so 300 measurements were performed in each group. All collected DLS data based on the percentage of the scattered light intensities are given in Table S5 (Supplementary Materials).

**Figure 4:**
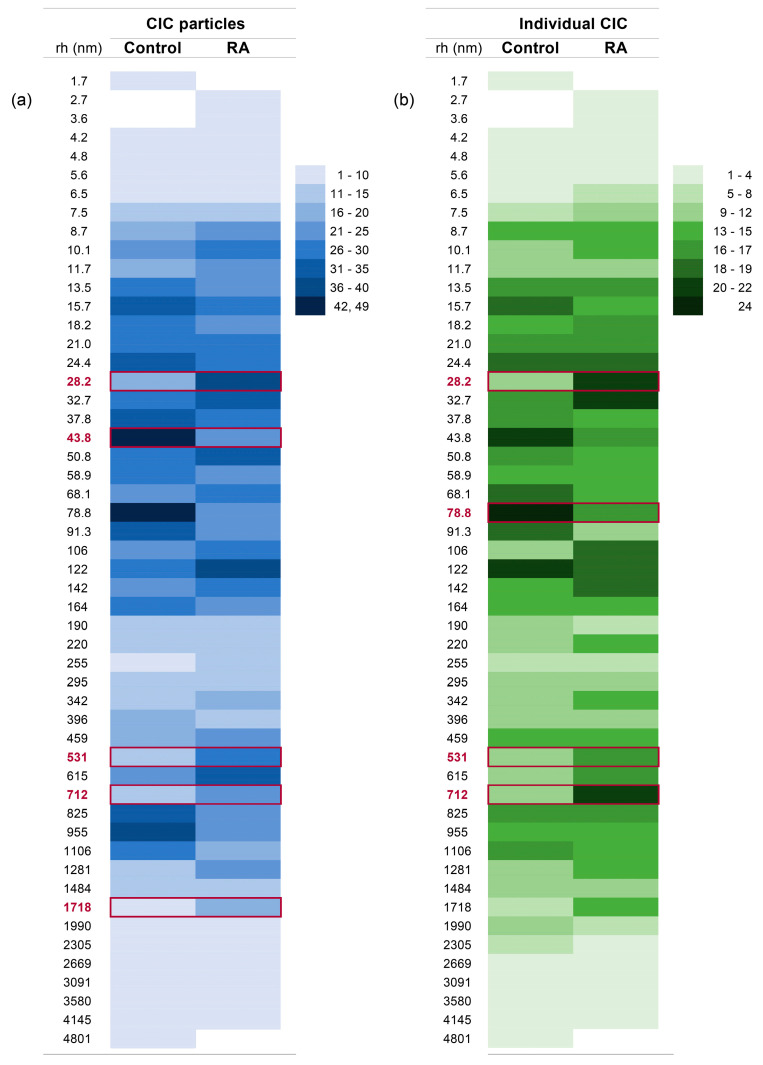
DLS analysis of CICs of the control group and RA patients group. (**a**) The total CIC particles’ size distribution of the analyzed groups (all measurements with ten repetitions); (**b**) the individual CIC rate of occurrence of specific particles. The distribution of particle size is based on light scattered intensity. The numerical data for this figure are given in the Supplementary Materials, Table S6a,b. In red squares: particles with different rates of occurrence in RA and control CICs.

The distribution of total recorded CIC particles by their hydrodynamic radii in the RA and the control group is displayed in Figure 4a. A total number of 938 particles in 30 RA CICs and 944 particles in control CICs was detected by DLS. In both groups, 50 different types of particles, distinguished by their average hydrodynamic radii, were detected. In the RA group, we detected 31 ± 7 (20–46 range; median 32) particles per individual CIC, and this did not differ from the control group 31 ± 6 (16–44 range; median 32). The most frequent particles in the CICs of RA patients and the CICs of control individuals were those with a hydrodynamic radius of 122 nm (recorded 39 times) and 78.8 nm (recorded 49 times), respectively. In both groups, the particles clustered around two size groups: the first with a radius range of 7.5–164 nm (606 and 594 particles in control and RA group) and the second with a radius range of 342–1718 nm (229 and 236 particles in control and RA group). The distribution of these particles between the groups was not the same, and the Mann–Whitney test showed that there was a significant increase in the frequency of particles with a radius of 28.2, 531, 712, and 1718 nm in the CICs of the RA group, compared to the CICs of the control group. These particles’ concomitant presence was observed in only four RA CICs (CIC RA9, RA16, RA18, and RA19; Supplementary Materials, Table S4). Using the same statistical test, a significantly higher number of particles with a radius of 43.8 nm was detected in control CICs than in RA CICs.

Using Fisher’s exact test to determine the number of individuals having CICs with particles of a specific size, we showed that there was a significantly higher number of individuals in the RA group compared to the control group whose CICs contained particles with a radius of 28.2, 531, and 712 nm (Figure 4b; Supplementary Materials, Table S6). The same test showed that only CICs containing particles with a radius of 78.8 nm occurred more often in individuals in the control group than in the RA group (Figure 4b; Supplementary Materials, Table S6).

### 2.3. The Effect of Colloidal Particle Size on the IgA, IgG, and IgM Level in CICs of RA Patients and Healthy Individuals

Following the determination of the level of IgA, IgG, and IgM within CICs (Figure 3), our investigation explored the association between CIC particles’ size distribution and immunoglobulin concentrations.

Figure 4 shows a statistically significant distinction between the control and RA groups in the distribution of particles with sizes of 28.8, 43.8, 78.8, 531, 712, and 1718 nm. Among them, the particles with sizes of 43.8 and 78.8 nm exhibited higher prevalence within the control CICs, and the particles with sizes of 28.8, 531, 712, and 1718 nm exhibited higher prevalence within RA CICs.

**Figure 5:**
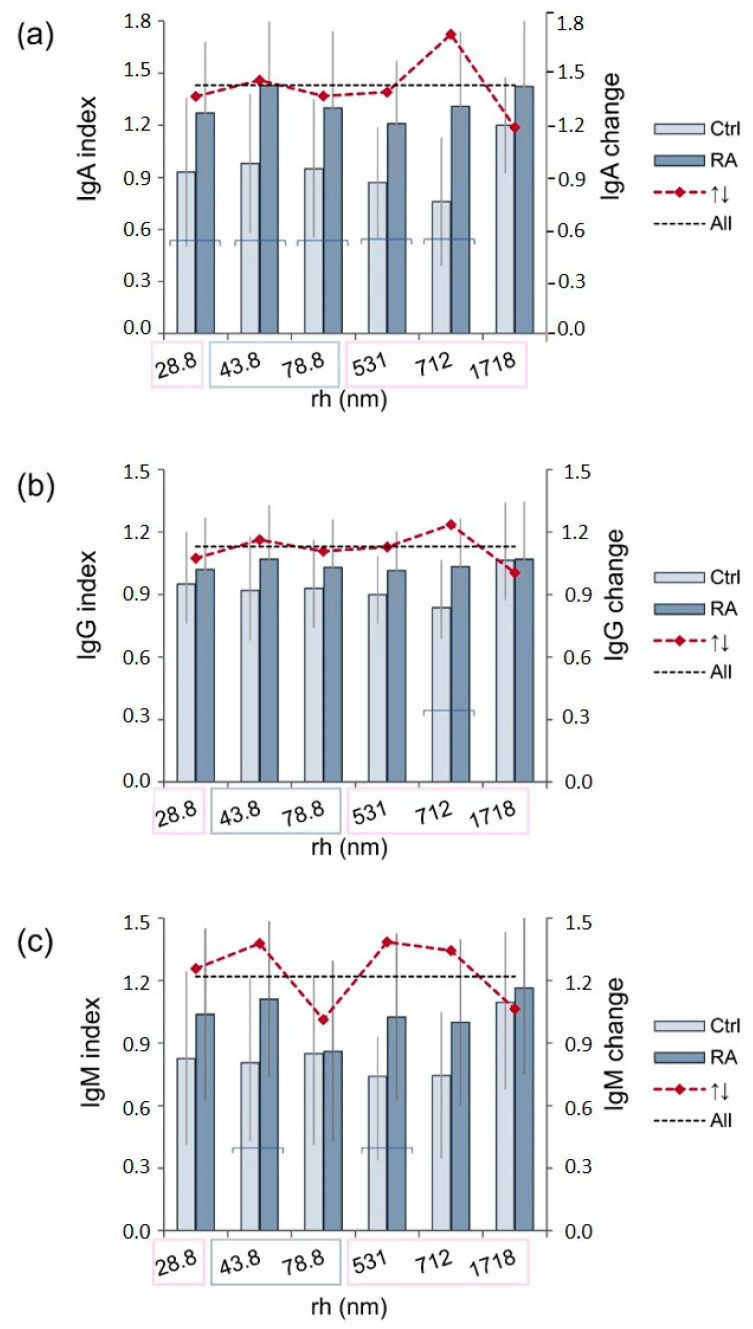
The relative content of IgA (**a**), IgG (**b**), and IgM (**c**) in CIC-containing particles was significantly differently expressed between RA and control CIC. The distribution of particle size is based on light scattered intensity. Immunoglobulin levels are determined by dot blot. Data are mean ± SD. Pink squares show particles which exhibited higher prevalence within RA CICs; blue squares show particles which showed higher prevalence within the control CICs. The ↑↓change in the IgA, IgG, and IgM level represents the ratio of mean values of the corresponding immunoglobulin class level in RA and control CICs. All—represents the increase in IgA, IgG, and IgM levels in all analyzed RA CICs compared to all control CICs. A statistically significant difference in immunoglobulin level between RA and control CICs is shown with horizontal bars. The numerical data for this figure are given in Supplementary Materials, Table S7a–c.

The CICs containing these particles were found in both RA and control groups, and the concentrations of immunoglobulin classes expressed within these CICs were compared (Figure 5). The IgA level was significantly higher in RA CICs containing any of the particles that were found to be expressed differently between the RA and the control group, except for the 1718 nm particle. The IgG increase was detected only in RA CICs containing 712 nm particles, while IgM was increased in RA CICs containing 43.8 or 712 nm particles.

**Figure 6:**
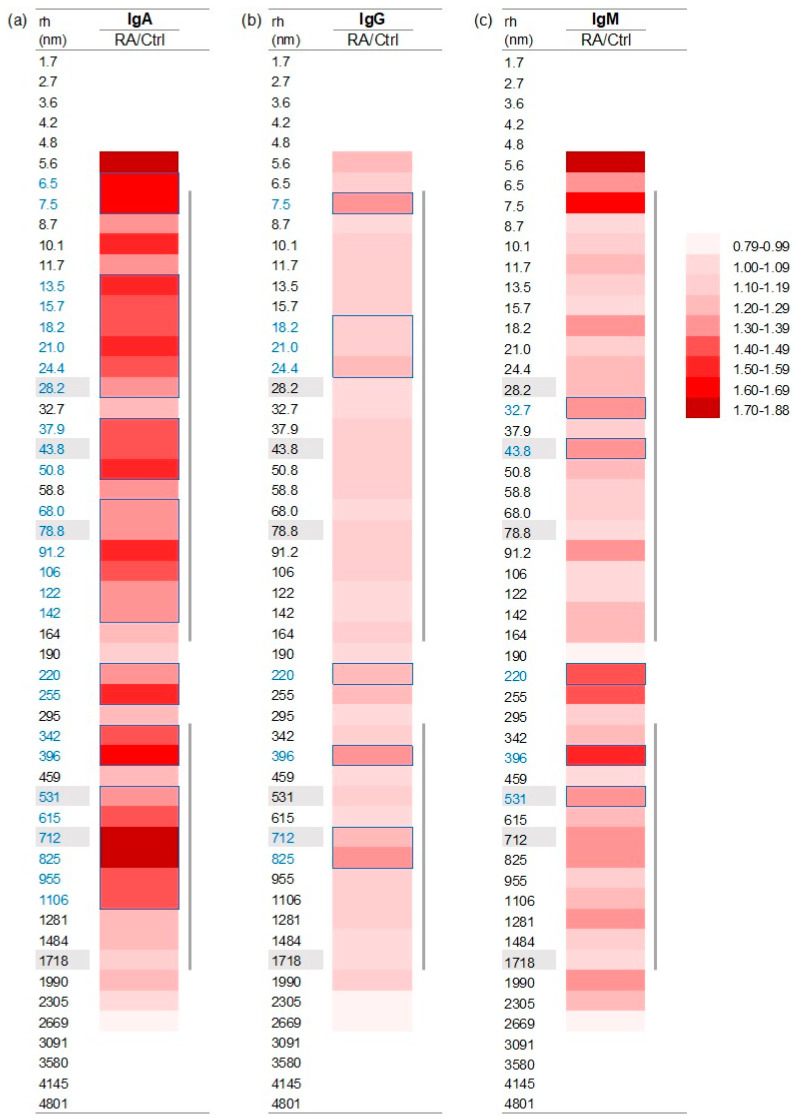
Relative content of IgA (**a**), IgG (**b**), and IgM (**c**) in RA and control CICs: distribution dependent on the CIC particle size. Particle size distribution is based on light scattered intensity. Immunoglobulin levels are determined by dot blot. Data are the ratio of mean values of IgA, IgG, and IgM in RA and control CICs. Numbers in blue and blue rectangles indicate a significant difference in IgA (a), IgG (b), or IgM (c) in RA compared to control CICs. Numbers shown with a grey background are particles differently expressed in RA and control CICs. Grey vertical lines: two main clusters of CIC particles. The numerical data for this figure are given in Supplementary Materials, Table S7a–c.

We noticed that the changes in the CICs’ immunoglobulin levels in RA CICs did not match the pattern of particle distribution, showing various expressions between the control and the RA groups. For example, the level of IgA in RA CICs containing 43.8 and 78.8 nm particles was higher than in the control group. Therefore, we decided to analyze the difference in immunoglobulin levels between RA and control CICs for particles that were detected in sufficient numbers for statistical analysis (43 particles, with a radius from 5.6 nm to 2669 nm). The data shown in Figure 6 and Table S7a–c (Supplementary Materials) demonstrate that a significantly increased level of IgA in RA compared to control CICs was associated with 28 particles. Among these 28 particles, an increased concentration of IgA of more than 50% was detected within the particles with radii of 6.5, 7.5, 13.5, 21.0, 50.8, 91.2, 225, 396, 712, 812, and 1106 nm. A significantly increased level of IgG in RA compared with control CICs was associated with eight particle sizes (7.5, 18.2, 21.0, 24.0, 220, 396, 712, and 812 nm). In contrast, the increased level of IgM was associated with particles having a radius of 32.7, 43.8, 220, 396, 531, and 1281 nm. The concomitant increase in the level of all three immunoglobulin classes in RA compared to the control was associated only with CICs containing 220 and 396 nm particles.

The number of investigated RA patients was insufficient to confirm the link of the presence of serum RFs or ACPAs with the specific particle size in the CICs of these patients. However, a preliminary statistical analysis showed that among ACPA+RF+ RA patients, compared to seronegative ones, there was a higher number of individuals with CICs containing 32.7 nm, and 1218 nm, and 1718 nm particles and a lower number of individuals with CICs containing 396 nm particles (Supplementary Materials Table S8). Interestingly, the level of any of the immunoglobulin classes did not differ between RA CICs with and without these particles.

## 3. Discussion

Rheumatoid arthritis is an autoimmune, systemic inflammatory disease of connective tissue [19]. The causative agents of RA are mainly unknown. It is believed that it could be triggered by chronic inflammation induced by RA-associated microorganisms in susceptible individuals, even a decade before the onset of symptoms [20].

ICs are potent inducers of local and systemic RA inflammation, but their precise role in RA pathogenesis still needs to be clarified. CICs and ICs in synovial fluid are likely to contribute to the pathogenesis of RA through the activation of the complement cascade, activation of immune cells via Fc receptors, direct destruction of cartilage, and production of tumor necrosis factor and other proinflammatory mediators in synovial tissues [21]. Studies have shown that both patients’ synovial fluid and serum can be used for experimentation on ICs. However, collecting synovial fluid is invasive and burdensome and is not commonly used for diagnostic purposes. In our previous study, we successfully demonstrated the feasibility of DLS for the biophysical analysis of pooled ICs isolated from the serum of patients with RA [15]. This study aimed to measure the size of colloidal particles found in the sera of CICs of individual RA patients using DLS. Additionally, this study aimed to investigate whether the specific immunoglobulin isotypes in isolated CICs and the presence of ACPAs and RFs in peripheral blood were associated with particular sizes of CIC colloidal particles.

The quantity and architecture of ICs are dependent on the concentration of their constituents [10]. Data on the association of RA with immunoglobulin classes/subclasses are contradictory [16,17], so we intended to determine specific IgG, IgA, and IgM levels in the ICs of RA patients. Also, RA ICs contain two main categories of autoantibodies, RFs and ACPAs, which can be detected in both synovial fluid and peripheral blood [3,4]. Importantly, positive results for ACPAs and RFs in patients’ sera are considered as part of the definitive diagnosis for RA [22]. These autoantibodies are also believed to be valuable biomarkers in predicting the clinical course of the disease; nevertheless, despite extensive research, the literature is still conflicting [23]. In a cross-sectional study, Aletaha et al. found that ACPA-positive patients had similar or lower disease activity than ACPA-negative patients, whether RFs were present or not [24]. Similarly, Miriovsky et al. discovered that a higher ACPA concentration was linked to a greater chance of remission in ACPA-positive/RF-negative patients [25]. However, in ACPA-negative/RF-positive patients, a higher RF concentration was associated with a decreased likelihood of remission, although this was not significant. Despite RF positivity being a significant predictor of structural joint damage, Mottönen et al. demonstrated that it was not a significant predictor of disease remission [26]. Interestingly, Pope et al. (2018) showed that patients with RA positive for both ACPAs and RFs responded better and faster to antirheumatic medications [23]. Still, other investigators [27,28,29] came to different conclusions regarding the disease activity and RF/ACPA positivity.

It is known that RFs and ACPAs can recruit complement via both the classical and alternative pathways but not via the lectin pathway [30]. Elevated levels of complement activation products and increased consumption of C3 and C4 result in their reduced level in the peripheral blood and synovial fluid of RA patients [31]. The reduced complement protein level in RA reduces ICs’ solubility, influences their size, and thus acts in favor of IC deposition in joint tissue [32]. Both ACPAs and RFs are proven to form ICs in the peripheral blood and inflamed joints of RA patients [3,4], where they are involved in the activation of the complement cascade in RA synovial tissue [33]. Nevertheless, opposite to the previously mentioned link to disease activity, their relation to IC architecture, such as IC size and constituent isotypes in RA patients, has yet to be comprehensively studied.

Findings from the present study suggest no significant differences in the level of CICs among the RA and the control group. Furthermore, the presence of ACPAs and RFs did not impact the CIC level within the RA group. Within the RA group, a negative correlation was demonstrated between the levels of CICs and ACPAs; it should be noted that since PEG precipitation is a selective method [34], there is a possibility that PEG may not precipitate ACPAs. Additionally, it should be emphasized that this method yields only one category of ICs (only high- (>19S) and intermediate- (8 to 19S) molecular-weight ICs [34] described as less physicochemically stable [35]. Furthermore, the RF and ACPA positivity relied on only one test used in the clinics that provided us with the samples (described in the Material and Methods, Section 4.2.)

The sera of patients with RA were enriched with IgG and IgA isotypes of ACPAs, but IgM ACPAs may also be present [36]. This contrasts with RF isotypes, where different types (IgM > IgA > IgG) were identified [37,38]. There was also an early increase in the frequency and concentration of IgG, particularly IgG1 subclasses in RA patients or those at risk of developing the disease. Preclinical samples showed a predominant production of IgA-isotype ACPAs among subjects at risk for RA [37]. Our study showed that the serum RF titer correlated with the level of all classes of immunoglobulins, while the serum ACPA titer correlated only with the level of CIC IgA in RA patients. At this moment, we can only speculate that such results might indicate that ACPAs form PEG-precipitable complexes with IgA.

Although RFs are predominately of the IgM isotype, the switch of RF isotype and high titers of IgM and IgA RFs are characteristic of RA [39]. IgM or IgA RF-ACPA- immune complexes contribute to RA synovitis by activating complement, FcR triggering and activation of macrophages and neutrophils, and enhancing osteoclast differentiation [37]. It is important to note that B cells that express membrane RFs can bind and internalize ACPAs, which leads to autoantigen presentation, T cell activation and maturation, expansion of ACPA-secreting B cells, and epitope spreading [38]. In contrast to IgM and IgA RFs, the role of IgG RFs in the pathogenesis of RA remains poorly understood. We can only assume that IgG RFs might contribute to RA pathogenesis by FcR-mediated antibody enhancement [40], but data strongly confirm that this role of IgG RFs does not exist. A deeper study of the role of IgG RFs is necessary, knowing that although IgM RFs are the most common RF isotype among RA in patients of Caucasian origin, IgG RFs represent the dominating RF isotype in Asian populations (irrespective of their ethnicity) [41].

In this study, RFs were detected by nephelometric methods, described as a method that is highly sensitive and widely used in clinical practice [42]. We are aware that the absence of data on serum and CIC RF isotypes (as well as ACPA isotypes) represents a significant drawback of our study, so we can only speculate on the origin of the CIC immunoglobulin detected. However, a positive correlation between the CIC level and CIC IgM, but not IgG or IgA in RA, reflects the selective accumulation of IgM in PEG-precipitable CICs. Whether these IgM molecules represent RFs is not clear at this moment, especially considering the observed absence of correlation between the titer of serum RFs and the level of CICs. Our findings showing that an increased level of CIC IgG, but not CIC IgA or IgM, is associated with higher serum ACPA and RF titer and might indicate that PEG precipitates IgG ACPA-RF immunocomplexes.

The very first study by Mageed et al. (1991) [3] on the characterization of the size and composition of CICs in RA patients using sucrose density gradient ultracentrifugation gave pilot results regarding the link between CIC size and their composition and some insights into the diagnostic importance of CIC analysis. By analyzing the serum of RA patients, Mageed et al. (1991) [3] demonstrated the presence of ICs of 22S size by analytical ultracentrifugation, leading to the suggestion that these might consist of one molecule of IgM RF bound to five molecules of IgG [3].

Given the demonstrated significance of the immunoglobulins as the constituents of CICs, as well as the size of CICs in the pathogenesis and diagnosis of RA, in this study, we aimed to extend our understanding of the size of CICs and explore potential associations between CIC size analyzed individually and the classes of immunoglobulins present within them.

Along with the technology development, György and co-workers (2011) [14] and Djukic et al. (2023) [15] demonstrated that the DLS method could be used for relatively quick analysis of the size of colloidal particles forming ICs in RA patients’ pooled samples of synovial fluid and peripheral blood serum ICs, respectively [14,15]. György et al. (2011) [14] reported that using DLS, it was possible to identify particles of 4, 210, and 2000 nm in size (IgG ICs) or particles of 12, 40, 210, and 2100 nm in size (IgM ICs) in aggregated samples of synovial fluid from patients with RA [14]. On the other hand, our investigation has demonstrated that the IgG increase was detected in RA CICs containing 712 nm particles. An increased level of IgM was observed in RA CICs containing 43.8 or 712 nm particles. Discrepancies in these findings may arise from variations in CIC origin and methodological approaches used [43,44]. Namely, we isolated CICs by PEG precipitation from the serum of RA patients. At the same time, in the study of György et al. (2011) [14], IgG and IgM affinity-isolated ICs from synovial fluid were used.

Although the hydrodynamic radii of immunoglobulin monomers (IgG—5.29 nm; IgA—6.50 nm; IgM—12.6 nm), as well as their proteolytic fragments (F(ab′)2—4.48 nm; Fc—3.19 nm; Fab—2.19 nm), are known [45], we cannot predict the molecular composition of dominant RA CIC colloidal particles identified in our study. The main reasons could be (1) that the buffer systems used, pH, and interaction with antigens induce conformation and hydrodynamic radii changes in immunoglobulin monomers [43,44] and (2) that the radii of the formed immunoglobulin (or any other protein) aggregates are not equal to the sum of the diameters of the monomers forming the aggregates [46].

By analyzing individual samples of serum CICs from RA patients by DLS, we mapped the dominant CIC particle sizes, which generally clustered around two broad-size peaks. This was in accordance with our previous study, where we measured pooled CIC samples [15]. Additionally, in this study, CIC-containing particles significantly differed in size between the studied groups and were correlated with immunoglobulin concentrations to ascertain any potential associations. The results indicated a statistically significant increase in IgA level within RA CICs compared to the control CICs, accompanied by variations in their respective sizes. This result could have importance for diagnostic purposes, considering the proven osteoclast-mediated bone resorption through IgA-FcαRI interaction and NET formation by IgA-containing ICs [11,12]. However, it merits further investigation to correlate the level of disease activity and particular features with CIC size, as suggested [3] before rapid and effective technologies like DLS. We acknowledge the limitation of the small sample size in each group, which we identify as a weakness of this study. Larger-scale studies are necessary for more accurate and robust results to use CIC size as a criterion in RA disease diagnosis or monitoring.

## 4. Materials and Methods

### 4.1. Participants

All participants in this study provided written consent before their peripheral blood samples were collected. We followed the Code of Ethics established by the World Medical Association (Declaration of Helsinki) and the approvals of the Ethics Committees of School of Dentistry, University of Belgrade (Ethical approval No 36/7; 12 April 2017).

In this study, 30 RA patients participated as in-patients who were cured at the Institute of Rheumatology (Belgrade, Serbia). The patient age, gender, RF and ACPA levels, SER, concentration of CRP, CIC level, disease activity score (DAS) 28, and disease-specific therapy received at the time of the blood collection are given in Table S1, Supplementary Materials. None of the RA patients were treated with therapeutic monoclonal antibodies. The peripheral blood samples of the control group were collected at the Clinic of Periodontology, School of Dental Medicine, University of Belgrade. The control group comprised 30 healthy middle-aged and older adults, representing RA age-matched control. The control group participants had no documented systemic diseases or acute infections one month before the blood sampling. Their age and gender are also given in Table S1 (Supplementary Materials).

### 4.2. Determination of Serum RF and ACPA Titer

RF titer was measured with a RF II turbidimetric assay purchased from Mindray, China. According to the manufacturer, the assay’s cut-off value is 14. RF isotype was not determined.

ACPAs, i.e., IgG antibodies to citrullinated peptides, were detected with ELISA produced by EUROIMMUN Schweiz AG, Switzerland. The target antigen used in the assay is a cyclic citrullinated peptide (CCP2). The assay’s cut-off value is 5 RU/mL.

### 4.3. Isolation of CICs and Determination of CICs and the CIC Immunoglobulin Level

Blood was drawn from the antecubital vein into 9 mL silicone-coated plastic tubes with no anticoagulant (BD Vacutainer^®^ Venous Blood Collection, BD Diagnostics, NJ, USA). Blood serum was separated after spontaneous coagulation for 24 h at 37 °C and 10 min centrifugation at 22 °C, at 1257× *g*.

The CICs were isolated by polyethylene glycol (PEG) precipitation of serum proteins with a final PEG concentration of 3% (*w*/*v*). After redissolving in PBS, the CIC samples were aliquoted and stored at −20 °C. After a maximum of one year of storage at −20 °C, the CIC samples were thawed, and the absorbance of every individual CIC was measured on an Ultrospec 3300 pro spectrophotometer (Amersham Bioscience, Uppsala, Sweden) at 350 nm (OD350) [15]. After the measurement, each CIC sample was analyzed by DLS as described in the following Section 4.4.

The relative content of immunoglobulin classes (i.e., immunoglobulin index) for every individual CIC was assessed by dot blot protocols from our previous papers [47,48,49]. The CIC IgA, IgG, and IgM levels were determined using peroxidase-conjugated goat anti-human IgA, IgG, and IgM antibodies (Boster Biological Technology Co., Ltd., Pleasanton, CA, USA). The IgA, IgG, or IgM index represents the intensity of IgA, IgG, or IgM dot blot signal normalized to the assay control (pooled healthy human serum; 83 g/L total proteins) diluted to the protein concentration of 2.1 mg/mL, which corresponded to the concentration of 3% PEG-precipitated serum proteins (our laboratory standard). The concentration of IgG, IgA, and IgM in pooled sera was determined by immunonephelometry [15], and in diluted sera, the concentration was 0.20, 0.04, and 0.02 g/L, respectively.

### 4.4. Size of CICs

The average particle size of isolated CICs was determined using DLS, as described in Djukic et al. (2023) [15]. In short, DLS measures the time-dependent fluctuation of the light scattered by molecules in a solution to determine the translational diffusion coefficient and, subsequently, the hydrodynamic radius from the Stokes–Einstein equation. In this study, we utilized a Zetasizer Nano ZS provided by Malvern Instruments (U.K.) to carry out the measurements. The device functioned at a wavelength of 633 nm and was equipped with a He-Ne laser. The measurements were conducted at a scattering angle of 173° and a steady temperature of 25.0 ± 0.1 °C, using disposable polystyrene cuvettes. Each sample’s measurements were repeated ten times.

### 4.5. Statistical Analysis

Statistical analysis was performed using SPSS v.25 software. A descriptive statistics calculation was separately performed for the study participants’ age, analyzed clinical parameters (blood serum RF, ACPA, CIC, and CRP level, SER and DAS score), and the CIC immunoglobulin levels. The normality of distribution was determined with the Shapiro–Wilk test. The association between two parameters was determined, depending on the normality of their distributions, by Pearson product-moment correlation or Spearman rank-order correlation test. To evaluate the differences between analyzed groups in the level of CICs and the level of CIC immunoglobulins, we employed either a two-tailed t-test or the Mann–Whitney U test, depending on the distribution’s normality. *p*-values < 0.05 were considered significant.

Total DSC data were analyzed with the Mann–Whitney U test with the aim to evaluate the difference between the RA and the control group in the total number of particles with a specific size. Fisher’s exact test was used to determine the difference between the RA and control group in the number of individuals with CICs with particles of particular sizes. *p*-values < 0.10 were considered significant.

## 5. Conclusions

Our study aimed to determine if the presence of RFs and ACPAs in sera and the levels of IgA, IgG, and IgM in CICs are associated with the hydrodynamic sizes of particles determined by DLS in PEG-precipitable CICs of individual RA patients.

We found a significant correlation between the level of CICs and CIC IgM relative content in RA. Additionally, the level of all CIC immunoglobulin classes showed a strong correlation with the level of serum RFs, while only CIC IgA in RA was correlated with ACPA titer.

We detected 50 different particles, distinguished by their size, in both control and RA CICs. An increased level of IgA in RA CIC was associated with more than 50% of the existing CICs having different sizes compared to control CICs. We found that CIC particles of 28.8, 43.8, 78.8, 531, 712, and 1718 nm in size were significantly differently expressed between the RA and the control group. The elevated IgA in RA patients was linked with all these particles, except the 1718 nm ones. The IgG increase was only detected within 712 nm RA CIC particles, while IgM was increased in 43.8 or 712 nm RA CIC particles.

For the first time, this study provided an insight into the link between hydrodynamic size and the immunoglobulins of CICs in RA patients. It is imperative to conduct larger-scale studies to enhance the reliability and precision of CIC size determined by DLS as a criterion in RA diagnosis or monitoring. Furthermore, such studies should be followed by the introduction of advanced biochemical, physicochemical, and immunochemical methods for the analysis of the molecular composition of CICs.

## Figures and Tables

**Table 1:** Demographic and clinical data of the study participants.

		RA	
	All	ACPA+ RF+	ACPA-RF-
No	30	20	6
M/F	5/25	4/15	1/5
	62 ± 11	64 ± 11	62 ± 6
Age (year)	64	67	62
	(26–76)	(26–76)	(49–70)
	82 ± 85	122 ± 78	/
RF (IU/mL)	69	91	/
	(0–361)	(46–361)	/
	260 ± 292	364 ± 304	/
ACPA (RU/mL)	167	298	/
	(0–1180)	(27–1180)	/
	29 ± 18	34 ± 19	14 ± 8
SER (mm/h)	28	28	13 *
	(4–68)	(4–68)	(6–32)
	13.6 ± 10.6	15.4 ± 11.7	8.9 ± 5.0
CRP (mg/L)	10.3	12.20	7.6
	(0.0–38.7)	(0.0–38.7)	(4.1–18.9)
	3.97 ± 1.10	4.17 ± 1.33	3.38 ± 0.34
DAS	4.07	4.44	3.25
	(1.25–5.65)	(1.25–5.65)	(3.06–3.96)
		**Control**	
No		30	
M/F		11/9	
		55 ± 14	
Age (year)		59	
		(22–71)	

Data: mean ± SD, median, and minimum-to-maximum range (in the parenthesis); (*)—significant differences between ACPA+RF+ and ACPA-RF- patients in SER (*p* = 0.031).

## Data Availability

The original data are given in Supplementary Materials Tables S1 and S4.

## Supplementary Materials

The following supporting information can be downloaded at https://www.mdpi.com/article/10.3390/ijms25063138/s1.

## Abbreviations

ACPAs	antibodies against citrullinated peptides
CCP2	cyclic citrullinated peptide
CICs	circulating immune complexes
CRP	C reactive protein
DAS	disease activity score
DLS	dynamic light scattering
DMARDs	disease-modifying antirheumatic drugs
ICs	immune complexes
ODs	optical densities
PEG	polyethylene glycol
RA	rheumatoid arthritis
RF	rheumatoid factor
SER	erythrocyte sedimentation rate
NET	neutrophil extracellular traps

## References

[1] Chang M.H., Nigrovic P.A. (2019). Antibody-Dependent and -Independent Mechanisms of Inflammatory Arthritis. JCI Insight.

[2] Maibom-Thomsen S.L., Trier N.H., Holm B.E., Hansen K.B., Rasmussen M.I., Chailyan A., Marcatili P., Højrup P., Houen G. (2019). Immunoglobulin G Structure and Rheumatoid Factor Epitopes. PLoS ONE.

[3] Mageed R.A., Kirwan J.R., Thompson P.W., McCarthy D.A., Holborow E.J. (1991). Characterisation of the Size and Composition of Circulating Immune Complexes in Patients with Rheumatoid Arthritis. Ann. Rheum. Dis..

[4] Sohrabian A., Mathsson-Alm L., Hansson M., Knight A., Lysholm J., Cornillet M., Skriner K., Serre G., Larsson A., Weitoft T. (2018). Number of Individual ACPA Reactivities in Synovial Fluid Immune Complexes, but Not Serum Anti-CCP2 Levels, Associate with Inflammation and Joint Destruction in Rheumatoid Arthritis. Ann. Rheum. Dis..

[5] Luthra H.S., McDuffie F.C., Hunder G.G., Samayoa E.A. (1975). Immune Complexes in Sera and Synovial Fluids of Patients with Rheumatoid Arthritis. Radioimmunoassay with Monoclonal Rheumatoid Factor. J. Clin. Investig..

[6] Zubler R.H., Nydegger U., Perrin L.H., Fehr K., McCormick J., Lambert P.H., Miescher P.A. (1976). Circulating and Intra Articular Immune Complexes in Patients with Rheumatoid Arthritis. Correlation of 125I-C1q Binding Activity with Clinical and Biological Features of the Disease. J. Clin. Investig..

[7] Reeback J.S., Silman A.J., Holborow E.J., Maini R.N., Hay F.C. (1985). Circulating Immune Complexes and Rheumatoid Arthritis: A Comparison of Different Assay Methods and Their Early Predictive Value for Disease Activity and Outcome. Ann. Rheum. Dis..

[8] Antes U., Heinz H.P., Schultz D., Brackertz D., Loos M. (1991). C1q-Bearing Immune Complexes Detected by a Monoclonal Antibody to Human C1q in Rheumatoid Arthritis Sera and Synovial Fluids. Rheumatol. Int..

[9] Michelin M.A., Crott L.S.P., Assis-Pandochi A.I., Coimbra T.M., Teixeira J.E., Barbosa J.E. (2002). Influence of the Electric Charge of the Antigen and the Immune Complex (IC) Lattice on the IC Activation of Human Complement. Int. J. Exp. Pathol..

[10] Negishi-Koga T., Gober H.J., Sumiya E., Komatsu N., Okamoto K., Sawa S., Suematsu A., Suda T., Sato K., Takai T. (2015). Immune Complexes Regulate Bone Metabolism through FcRγ Signalling. Nat. Commun..

[11] Breedveld A.C., van Gool M.M.J., van Delft M.A.M., van der Laken C.J., de Vries T.J., Jansen I.D.C., van Egmond M. (2021). IgA Immune Complexes Induce Osteoclast-Mediated Bone Resorption. Front. Immunol..

[12] Gimpel A.K., Maccataio A., Unterweger H., Sokolova M.V., Schett G., Steffen U. (2022). IgA Complexes Induce Neutrophil Extracellular Trap Formation More Potently Than IgG Complexes. Front. Immunol..

[13] Konig M.F., Abusleme L., Reinholdt J., Palmer R.J., Teles R.P., Sampson K., Rosen A., Nigrovic P.A., Sokolove J., Giles J.T. (2016). Aggregatibacter Actinomycetemcomitans-Induced Hypercitrullination Links Periodontal Infection to Autoimmunity in Rheumatoid Arthritis. Sci. Transl. Med..

[14] György B., Módos K., Pállinger É., Pálóczi K., Pásztói M., Misják P., Deli M.A., Sipos Á., Szalai A., Voszka I. (2011). Detection and Isolation of Cell-Derived Microparticles Are Compromised by Protein Complexes Resulting from Shared Biophysical Parameters. Blood.

[15] Djukić T., Drvenica I., Kovačić M., Minić R., Vučetić D., Majerič D., Šefik-Bukilica M., Savić O., Bugarski B., Ilić V. (2023). Dynamic Light Scattering Analysis of Immune Complexes in Sera of Rheumatoid Arthritis Patients. Anal. Biochem..

[16] Yu K.H., Chan T.M., Tsai P.H., Cheng C.H., Chang P.Y. (2015). Diagnostic Performance of Serum IgG4 Levels in Patients with IgG4-Related Disease. Medicine.

[17] Wang Y., Lloyd K.A., Melas I., Zhou D., Thyagarajan R., Lindqvist J., Hansson M., Svärd A., Mathsson-Alm L., Kastbom A. (2019). Rheumatoid Arthritis Patients Display B-Cell Dysregulation Already in the Naïve Repertoire Consistent with Defects in B-Cell Tolerance. Sci. Rep..

[18] Haville S., Deane K.D. (2022). Pre-RA: Can early diagnosis lead to prevention?. Best Pract. Res. Clin. Rheumatol..

[19] Zlatković-Švenda M.I., Stojanović R.M., Šipetić-Grujičić S., Guillemin F. (2013). Prevalence of rheumatoid arthritis in Serbia. Rheumatol. Int..

[20] Ge C., Holmdahl R. (2019). The structure, specificity and function of anti-citrullinated protein antibodies. Nat. Rev. Rheumatol..

[21] Ohyama K., Ueki Y., Kawakami A., Kishikawa N., Tamai M., Osaki M., Kuroda N. (2011). Immune complexome analysis of serum and its application in screening for immune complex antigens in rheumatoid arthritis. Clin. Chem..

[22] Aletaha D., Neogi T., Silman A.J., Funovits J., Felson D.T., Bingham C.O., Birnbaum N.S., Burmester G.R., Bykerk V.P., Cohen M.D. (2010). 2010 Rheumatoid Arthritis Classification Criteria: An American College of Rheumatology/European League Against Rheumatism Collaborative Initiative. Arthritis Rheum..

[23] Pope J.E., Movahedi M., Rampakakis E. (2019). Correction: ACPA and RF as Predictors of Sustained Clinical Remission in Patients with Rheumatoid Arthritis: Data from the Ontario Best Practices Research Initiative (OBRI). RMD Open.

[24] Aletaha D., Alasti F., Smolen J.S. (2015). Rheumatoid Factor, Not Antibodies against Citrullinated Proteins, Is Associated with Baseline Disease Activity in Rheumatoid Arthritis Clinical Trials. Arthritis Res. Ther..

[25] Miriovsky B.J., Michaud K., Thiele G.M., O’Dell J.R., Cannon G.W., Kerr G., Richards J.S., Johnson D., Caplan L., Reimold A. (2010). Anti-CCP Antibody and Rheumatoid Factor Concentrations Predict Greater Disease Activity in Men with Rheumatoid Arthritis. Ann. Rheum. Dis..

[26] Möttönen T., Paimela L., Leirisalo-Repo M., Kautiainen H., Ilonen J., Hannonen P. (1998). Only High Disease Activity and Positive Rheumatoid Factor Indicate Poor Prognosis in Patients with Early Rheumatoid Arthritis Treated with “sawtooth” Strategy. Ann. Rheum. Dis..

[27] van der Helm-van Mil A.H.M., Verpoort K.N., Breedveld F.C., Toes R.E.M., Huizinga T.W.J. (2005). Antibodies to Citrullinated Proteins and Differences in Clinical Progression of Rheumatoid Arthritis. Arthritis. Res. Ther..

[28] Zoto A., Selimi B. (2013). The Relationship of Rheumatoid Factor with Disease Activity in Patients with Rheumatoid Arthritis. Int. J. Sci. Res..

[29] Rönnelid J., Wick M.C., Lampa J., Lindblad S., Nordmark B., Klareskog L., Van Vollenhoven R.F. (2005). Longitudinal Analysis of Citrullinated Protein/Peptide Antibodies (Anti-CP) during 5 Year Follow up in Early Rheumatoid Arthritis: Anti-CP Status Predicts Worse Disease Activity and Greater Radiological Progression. Ann. Rheum. Dis..

[30] Trouw L.A., Haisma E.M., Levarht E.W.N., Van Der Woude D., Ioan-Facsinay A., Daha M.R., Huizinga T.W.J., Toes R.E. (2009). Anti-Cyclic Citrullinated Peptide Antibodies from Rheumatoid Arthritis Patients Activate Complement via Both the Classical and Alternative Pathways. Arthritis Rheum..

[31] Okroj M., Heinegård D., Holmdahl R., Blom A.M. (2007). Rheumatoid Arthritis and the Complement System. Ann. Med..

[32] Dayer E., Gerster J.C., Aguado M.T., Lambert P.H. (1983). Capacity to Solubilize Immune Complexes in Sera and Synovial Fluids from Patients with Rheumatoid Arthritis. Arthritis Rheum..

[33] Zhao X., Okeke N.L., Sharpe O., Batliwalla F.M., Lee A.T., Ho P.P., Tomooka B.H., Gregersen P.K., Robinson W.H. (2008). Circulating Immune Complexes Contain Citrullinated Fibrinogen in Rheumatoid Arthritis. Arthritis Res. Ther..

[34] Mellencamp M.A., Preheim L.C., McDonald T.L. (1987). Isolation and Characterization of Circulating Immune Complexes from Patients with Pneumococcal Pneumonia. Infect. Immun..

[35] Poulton T.A., Mooney N.A., Nineham L.J., Hay F.C. (1983). Characteristics of immune complexes detectable by two independent assays in gynaecological malignancies. Clin. Exp. Immunol..

[36] Ärlestig L., Mullazehi M., Kokkonen H., Rocklöv J., Rönnelid J., Dahlqvist S.R. (2011). Antibodies against cyclic citrullinated peptides of IgG, IgA and IgM isotype and rheumatoid factor of IgM and IgA isotype are increased in unaffected members of multicase rheumatoid arthritis families from northern Sweden. Ann. Rheum. Dis..

[37] Wu C.Y., Yang H.Y., Lai J.H. (2020). Anti-Citrullinated Protein Antibodies in Patients with Rheumatoid Arthritis: Biological Effects and Mechanisms of Immunopathogenesis. Int. J. Mol. Sci..

[38] Malström V., Grönwall C. (2018). The Parallel Worlds of ACPA-Positive and RF-Positive B Cells. Nat. Rev. Rheumatol..

[39] Volkov M., van Schie K.A., van der Woude D. (2020). Autoantibodies and B Cells: The ABC of rheumatoid arthritis pathophysiology. Immunol. Rev..

[40] Bournazos S., Gupta A., Ravetch J.V. (2020). The role of IgG Fc receptors in antibody-dependent enhancement. Nat. Rev. Immunol..

[41] Too C.L., Johan R. (2014). Increased IgG rheumatoid factor-positivity in the Asian rheumatoid arthritis patients irrespective of ethnicity. Open J. Rheumatol. Autoimmune Dis..

[42] Motta F., Bizzaro N., Giavarina D., Franceschini F., Infantino M., Palterer B., Sebastiani G.D., Selmi C. (2023). Rheumatoid factor isotypes in rheumatoid arthritis diagnosis and prognosis: A systematic review and meta-analysis. RMD Open.

[43] Gagnon P., Nian R., Lee J., Tan L., Latiff S.M., Lim C.L., Chuah C., Bi X., Yang Y., Zhang W. (2014). Nonspecific interactions of chromatin with immunoglobulin G and protein A, and their impact on purification performance. J. Chromatogr. A.

[44] Gagnon P., Nian R., Yang Y., Yang Q., Lim C.L. (2015). Non-immunospecific association of immunoglobulin G with chromatin during elution from protein A inflates host contamination, aggregate content, and antibody loss. J. Chromatogr. A.

[45] Armstrong J.K., Wenby R.B., Meiselman H.J., Fisher T.C. (2004). The hydrodynamic radii of macromolecules and their effect on red blood cell aggregation. Biophys. J..

[46] Djukic T., Drvenica I., Maslovaric I., Markovic D., Kovacic M., Minic R., Milanovic S., Bugarski B., Ilic V. Dynamic light scattering analysis of in vitro formed immune complexes, Cost Action CA21160, ML4 NGP, P34. Proceedings of the 1st Meeting on Machine Learning and Non-Globular Proteins.

[47] Brajović G., Stefanović G., Ilić V., Petrović S., Stefanović N., Nikolić-Jakoba N., Milošević-Jovčić N. (2010). Association of Fibronectin with Hypogalactosylated Immunoglobulin G in Gingival Crevicular Fluid in Periodontitis. J. Periodontol..

[48] Buač M., Mojsilović S., Mišić D., Vuković D., Savić O., Valčić O., Marković D., Gvozdić D., Ilić V., Fratrić N. (2016). Circulating Immune Complexes of Calves with Bronchopneumonia Modulate the Function of Peripheral Blood Leukocytes: In Vitro Evaluation. Res. Vet. Sci..

[49] Stefanović G., Marković D., Ilić V., Brajović G., Petrović S., Milošević-Jovčić N. (2006). Hypogalactosylation of Salivary and Gingival Fluid Immunoglobulin G in Patients with Advanced Periodontitis. J. Periodontol..

