# Supplementary material for: Exploring the Link between Hydrodynamic Size and Immunoglobulins of Circulating Immune Complexes in Rheumatoid Arthritis Patients
Source: Int J Mol Sci. 2024 Mar 8;25(6):3138. doi: 10.3390/ijms25063138 (PMC10970301; doi:10.3390/ijms25063138)
Supplement: Supplementary file 1 [file ijms-25-03138-s001.zip › ijms-2857184-supplementary/ijms-2857184-supplementary.pdf]

Supplementary material

Table S1 – Clinical data and CIC IgA. IgG and IgM relative content

Excel file: Supplementary material Table S1.xlsx

Table S2. The correlation between the RF and ACPA titre and between the titres and measured parameters of inflammation (SER and CRP) and disease activity in RA patients.

		r	p
**RF titre**	**ACPA titre**	**0.70**	**< 0.001**
RF titre	SER (mm/h)	0.19	0.31
	CRP (mg/L)	-0.13	0.51
	DAS28	0.10	0.65
ACPA titre	SER (mm/h)	0.10	0.58
	CRP (mg/L)	0.04	0.84
	DAS28	0.06	0.78

SER – the erythrocyte sedimentation rate (mm/hours);

CRP –the concentration of C reactive protein (mg/L);

DAS28 – disease activity score

Table S3. The correlation between the level of CIC and the CIC immunoglobulin content in control and RA group (a) the correlation between serum ACPA or RF level and the CIC level and the CIC immunoglobulin content in RA group (b) and the between the level of CIC level and CIC Ig contents with parameters of inflammation (SE and CRP) and disease activity in RA patients (c).

(a)			r	p				r	p
Control	CIC level	CIC IgA	0.68	< 0.001					
		CIC IgG	0.63	< 0.001					
		CIC IgM	0.48	0.007					
RA	CIC level	CIC IgA	0.27	n.s.	RA	CIC level	CIC IgA	0.07	n.s.
all		CIC IgG	0.29	n.s.	ACPA+RF+		CIC IgG	0.09	n.s.
		CIC IgM	0.65	< 0.001			CIC IgM	0.54	0.01
(b)			r	p				r	p
RA	ACPA	CIC level	-0.23	n.s.	RA	ACPA	CIC level	-0.62	0.003
all		CIC IgA	0.45	0.012	ACPA+RF+		CIC IgA	0.32	n.s.
		CIC IgG	0.29	n.s.			CIC IgG	0.26	n.s.
		CIC IgM	0.12	n.s.			CIC IgM	-0.17	n.s.
RA	RF	CIC level	0.09	n.s.	RA	RF	CIC level	-0.09	n.s.
all		CIC IgA	0.51	0.004	ACPA+RF+		CIC IgA	0.37	n.s.
		CIC IgG	0.37	0.046			CIC IgG	0.46	0.04
		CIC IgM	0.38	0.036			CIC IgM	0.18	n.s.
RA	ACPA	RF							
(c)									
RA	CIC level	CRP	0.36	n.s.					
all		SER	0.35	n.s.					
		DAS28	0.38	n.s.					

CIC level – OD_{350} level of redissolved PEG precipitated serum protein (PEG precipitable CIC);
r – correlation coefficient; significant corelation - $p < 0.05$. n.s. – non significant correlation. $p \geq 0.05$.
ACPA: serum titre of ACPAs; RF: serum titre of RF; SER – the erythrocyte sedimentation rate (mm/hours)
CRP – the concentration of C reactive protein (mg/L); DAS28 – disease activity score

Table S4. The association (a) between CIC immunoglobulin content and CIC level in RA and control CIC and (b) between CIC immunoglobulin content and serum ACPA and RF in RA.

**(a)**												
Ctrl CIC	IgM ↑	IgM ↓	*p*	IgA ↑	IgA ↓	*p*	IgG ↑	IgG ↓	*p*	Ig ↑	Ig ↑	*p*
	median value											
OD350 nm	0.503	0.285	*0.016*	0.540	0.285	*<0.001*	0.503	0.285	*0.007*	0.833	0.285	*0.002*
CIC IgA	1.07	0.54	*0.008*	0.98	0.58	*<0.001*	0.98	0.59	*0.023*	1.07	0.54	*<0.001*
CIC IgG	1.22	0.78	*<0.001*	1.23	0.55	*<0.001*	1.22	0.78	*<0.001*	1.27	0.55	*<0.001*
CIC IgM	1.06	0.78	*<0.001*	1.11	0.80	*0.003*	1.11	0.78	*0.014*	1.15	0.73	*<0.001*
RA CIC	IgM ↑	IgM ↓	*p*	IgA ↑	IgA ↓	*p*	IgG ↑	IgG ↓	*p*	Ig ↑	Ig ↑	*p*
	median value											
OD350 nm	0.621	0.312	*0.001*	0.549	0.352	*n.s.*	0.523	0.350	*n.s.*	0.536	0.322	*0.031*
CIC IgA	1.20	0.59	*n.s.*	1.20	0.68	*<0.001*	1.15	0.59	*0.002*	1.37	0.54	*0.002*
CIC IgG	1.34	1.13	*0.052*	1.56	1.01	*0.004*	1.55	1.07	*<0.001*	1.45	0.94	*<0.001*
CIC IgM	1.17	0.91	*<0.001*	1.17	0.88	*0.002*	1.17	0.87	*0.003*	1.18	0.87	*<0.001*
**(b)**												
RA CIC	IgM ↑	IgM ↓	*p*	IgA ↑	IgA ↓	*p*	IgG ↑	IgG ↓	*p*	Ig ↑	Ig ↑	*p*
	number of samples											
ACPA+RF+	12	8		13	7		11	9		6	4	
ACPA+RF-	0	3	*n.s.*	0	3	*n.s.*	1	2	*n.s.*	1	2	*n.s.*
ACPA-RF+	1	0		0	1		0	1		0	0	
ACPA-RF-	1	4		2	4		3	3		3	3	
RA CIC	IgM ↑	IgM ↓		IgA ↑	IgA ↓		IgG ↑	IgG ↓		Ig ↑	Ig ↑	*p*
	median value											
ACPA titer	236	304	*n.s.*	365	255	*n.s.*	439	113	*0.014*	439	220	*n.s.*
RF titer	108	81		99	89		128	72	*0.015*	122	69	

ACPA+RF+; ACPA+RF-. ACPA-RF+. ACPA-RF-: Subgroups of RA patients formed based on the presence of the serum ACPA or RF autoantibodies.

IgA↑. IgM ↑. IgG↑ - The CIC samples with the IgM. IgG or IgG level above the median value;
IgA ↓. IgM ↓. IgG - The CIC samples with the IgM. IgG or IgG level below the median value;
CIC Ig ↑ - CIC samples having all immunoglobulin classes above the median value;
CIC Ig ↓ - CIC samples having all immunoglobulin classes below the median value.

Table S5 –DLS data

Excel file: Supplementary material Table S5.xlsx

Sheet 1 DLS data for control CIC

Sheet 2 DLS data for RA CIC

Table S6 – DLS data analysis – Numeric data presented in Figure 4

(a)

	CIC particles		
	No	No	
**rh (nm)**	Control	RA	p
1.7	1		n.s.
2.7		1	n.s.
3.6		4	n.s.
4.2	1	1	n.s.
4.8	1	1	n.s.
5.6	4	5	n.s.
6.5	6	9	n.s.
7.5	11	11	n.s.
8.7	19	25	n.s.
10.1	23	28	n.s.
11.7	17	21	n.s.
13.5	28	23	n.s.
15.7	31	27	n.s.
18.2	29	23	n.s.
21.0	26	30	n.s.
24.4	35	30	n.s.
**28.2**	19	36	**0.019**
32.7	26	33	n.s.
37.8	35	27	n.s.
**43.8**	42	25	n.s.
50.8	30	32	n.s.
58.9	26	24	n.s.
68.1	25	26	n.s.
78.8	***49***	32	**0.095**
91.3	31	25	n.s.
106	25	29	n.s.
122	30	***39***	n.s.
142	23	26	n.s.
164	26	22	n.s.
190	13	12	n.s.
220	15	15	n.s.
255	10	11	n.s.
295	14	13	n.s.
342	13	16	n.s.
396	20	15	n.s.
459	17	21	n.s.
**531**	14	26	**0.067**
615	22	32	n.s.
**712**	15	24	**0.069**
825	35	24	n.s.
955	36	22	n.s.
1106	29	19	n.s.
1281	15	21	n.s.
1484	15	14	n.s.
**1718**	7	18	n.s.
1990	10	8	n.s.
2305	6	3	n.s.
2669	3	3	n.s.
3091	1	2	n.s.
3580	8	2	n.s.
4145	3	2	n.s.
4801	4		n.s.
**Max**	**49**	**39**	

(b)

	Individual CIC				
	Control		RA		
**rh (nm)**	w/o	with	w/o	with	p
1.7	29	1	30	0	n.s.
2.7	30	0	29	1	n.s.
3.6	30	0	26	4	n.s.
4.2	29	1	29	1	n.s.
4.8	29	1	29	1	n.s.
5.6	27	3	26	4	n.s.
6.5	26	4	24	6	n.s.
7.5	22	8	21	9	n.s.
8.7	17	13	16	14	n.s.
10.1	18	12	16	14	n.s.
11.7	18	12	18	12	n.s.
13.5	14	16	13	17	n.s.
15.7	11	19	15	15	n.s.
18.2	15	15	14	16	n.s.
21.0	14	16	14	16	n.s.
24.4	11	19	12	18	n.s.
**28.2**	20	10	10	***20***	**0.019**
**32.7**	13	17	10	***20***	n.s.
37.8	14	16	15	15	n.s.
43.8	8	22	14	16	n.s.
50.8	13	17	16	14	n.s.
58.8	15	15	15	15	n.s.
68.1	12	18	15	15	n.s.
**78.8**	6	**24**	13	17	**0.095**
91.3	12	18	18	12	n.s.
106	18	12	12	18	n.s.
122	10	20	11	19	n.s.
142	16	14	12	18	n.s.
164	16	14	15	15	n.s.
190	19	11	22	8	n.s.
220	20	10	16	14	n.s.
255	23	7	23	7	n.s.
295	19	11	20	10	n.s.
342	21	9	16	14	n.s.
396	19	11	18	12	n.s.
459	17	13	16	14	n.s.
**531**	21	9	13	17	**0.067**
615	18	12	13	17	n.s.
**712**	18	12	10	***20***	**0.069**
825	13	17	14	16	n.s.
955	15	15	16	14	n.s.
1106	13	17	17	13	n.s.
1281	18	12	15	15	n.s.
1484	19	11	20	10	n.s.
1718	23	7	17	13	n.s.
1990	21	9	23	7	n.s.
2305	25	5	27	3	n.s.
2669	27	3	27	3	n.s.
3091	29	1	29	1	n.s.
3580	26	4	28	2	n.s.
4145	27	3	28	2	n.s.
4801	27	3	30		n.s.
**Max**		**24**		**20**	

Table S7a – DLS analysis – Numeric data presented in Figure 6a_IgA

rh (nm)	Ctrl (n)	RA (n)	Ctrl IgA mean	Ctrl IgA SD	RA IgA mean	RA IgA SD	IgA RA/Ctrl	*p*
1.7	1	0	**1.41**					
2.7	0	1			**1.56**			
3.6	0	4			**1.54**	0.34		
4.2	1	1	**0.06**		**1.97**			
4.8	1	1	**0.87**		**0.87**			
5.6	3	4	**0.76**	0.49	**1.44**	0.46	**1.88**	*n.s*
6.5	4	7	**0.79**	0.49	**1.33**	0.32	**1.68**	*0.054*
7.5	8	9	**0.79**	0.46	**1.32**	0.40	**1.67**	*0.024*
8.7	13	14	**0.97**	0.50	**1.28**	0.35	**1.32**	*n.s*
10.1	12	14	**0.93**	0.43	**1.43**	0.43	**1.54**	*n.s*
11.7	12	12	**1.07**	0.37	**1.39**	0.44	**1.30**	*n.s*
13.5	16	17	**0.87**	0.30	**1.33**	0.42	**1.52**	*0.001*
15.7	19	15	**0.97**	0.41	**1.37**	0.46	**1.42**	*0.010*
18.2	15	16	**0.82**	0.35	**1.18**	0.33	**1.43**	*0.007*
21.0	17	16	**0.91**	0.40	**1.43**	0.39	**1.57**	*0.001*
24.4	20	18	**0.95**	0.45	**1.33**	0.40	**1.40**	*0.009*
28.2	10	20	**0.93**	0.43	**1.27**	0.41	**1.36**	*0.045*
32.7	18	20	**1.09**	0.49	**1.31**	0.40	**1.20**	*n.s*
37.9	16	15	**0.99**	0.48	**1.41**	0.41	**1.43**	*0.013*
43.8	22	16	**0.98**	0.43	**1.43**	0.40	**1.46**	*0.002*
50.8	17	14	**0.88**	0.38	**1.34**	0.35	**1.52**	*0.002*
58.8	15	16	**0.92**	0.43	**1.21**	0.40	**1.32**	*n.s*
68.0	18	16	**0.93**	0.40	**1.29**	0.39	**1.38**	*0.013*
78.8	24	18	**0.95**	0.39	**1.30**	0.44	**1.37**	*0.009*
91.2	18	13	**0.87**	0.37	**1.37**	0.47	**1.57**	*0.003*
106	12	18	**0.87**	0.38	**1.25**	0.46	**1.44**	*0.024*
122	20	19	**0.96**	0.47	**1.26**	0.45	**1.31**	*0.053*
142	14	18	**1.00**	0.48	**1.35**	0.41	**1.35**	*0.035*
164	14	15	**1.07**	0.49	**1.34**	0.40	**1.25**	*n.s*
190	11	8	**1.09**	0.35	**1.29**	0.32	**1.18**	*n.s*
220	10	14	**0.99**	0.41	**1.34**	0.34	**1.36**	*0.030*
255	7	7	**0.94**	0.36	**1.42**	0.45	**1.50**	*0.051*
295	11	10	**0.94**	0.43	**1.20**	0.30	**1.28**	*n.s*
342	9	14	**0.93**	0.43	**1.35**	0.37	**1.44**	*0.022*
396	11	12	**0.71**	0.49	**1.18**	0.33	**1.67**	*0.012*
459	13	14	**1.01**	0.28	**1.23**	0.44	**1.22**	*n.s*
531	9	17	**0.87**	0.32	**1.21**	0.36	**1.39**	*0.027*
615	12	17	**0.93**	0.35	**1.31**	0.38	**1.41**	*0.009*
712	12	20	**0.76**	0.37	**1.31**	0.43	**1.74**	*0.001*
825	17	16	**0.76**	0.45	**1.30**	0.45	**1.70**	*0.002*
955	15	14	**0.92**	0.36	**1.33**	0.44	**1.45**	*0.010*
1106	17	13	**0.97**	0.46	**1.49**	0.42	**1.55**	*0.003*
1281	12	15	**1.11**	0.39	**1.35**	0.47	**1.22**	*n.s*
1484	11	10	**1.13**	0.37	**1.37**	0.29	**1.21**	*n.s*
1718	7	13	**1.20**	0.28	**1.42**	0.43	**1.19**	*n.s*
1990	9	7	**1.18**	0.49	**1.42**	0.43	**1.21**	*n.s*
2305	5	3	**1.25**	0.23	**1.32**	0.35	**1.06**	*n.s*
2669	3	3	**1.29**	0.23	**1.16**	0.44	**0.90**	*n.s*
3091	1	1	**1.22**		**1.67**		**1.37**	
3580	4	2	**1.25**	0.26	**1.38**			
4145	3	2	**1.16**	0.28	**1.09**			
4801	3	0	**0.67**	0.59				

Table S7b – DLS analysis – Numeric data presented in Figure 6b_IgG

rh (nm)	**Ctrl** (n)	**RA** (n)	**Ctrl IgG** **mean**	SD	**RA IgG** **mean**	SD	**IgG RA/Ctrl**	***p***
1.7	1	0	**1.29**					
2.7	0	1			**0.98**			
3.6	0	4			**0.97**	0.22		
4.2	1	1	**0.27**		**1.13**			
4.8	1	1	**0.87**		**0.81**			
5.6	3	4	**0.88**	0.27	**1.10**	0.20	**1.25**	*n.s*
6.5	4	7	**0.88**	0.18	**1.03**	0.11	**1.17**	*n.s*
7.5	8	9	**0.77**	0.24	**1.05**	0.28	**1.36**	*0.045*
8.7	13	14	**0.94**	0.28	**1.00**	0.17	**1.07**	*n.s*
10.1	12	14	**0.98**	0.19	**1.08**	0.26	**1.10**	*n.s*
11.7	12	12	**0.94**	0.16	**1.09**	0.24	**1.16**	*n.s*
13.5	16	17	**0.90**	0.17	**1.01**	0.21	**1.12**	*n.s*
15.7	19	15	**0.95**	0.22	**1.05**	0.26	**1.10**	*n.s*
18.2	15	16	**0.84**	0.18	**0.99**	0.17	**1.18**	*0.023*
21.0	17	16	**0.92**	0.20	**1.09**	0.23	**1.19**	*0.026*
24.4	20	18	**0.92**	0.23	**1.10**	0.22	**1.20**	*0.015*
28.2	10	20	**0.95**	0.19	**1.02**	0.25	**1.07**	*n.s*
32.7	18	20	**0.97**	0.24	**1.03**	0.25	**1.06**	*n.s*
37.9	16	15	**0.94**	0.25	**1.05**	0.24	**1.12**	*n.s*
43.8	22	16	**0.92**	0.24	**1.07**	0.26	**1.17**	*n.s*
50.8	17	14	**0.88**	0.24	**1.02**	0.24	**1.16**	*n.s*
58.8	15	16	**0.90**	0.22	**0.99**	0.25	**1.10**	*n.s*
68.0	18	16	**0.92**	0.22	**0.99**	0.24	**1.07**	*n.s*
78.8	24	18	**0.93**	0.19	**1.03**	0.23	**1.11**	*n.s*
91.2	18	13	**0.90**	0.21	**1.06**	0.27	**1.18**	*n.s*
106	12	18	**0.89**	0.18	**1.03**	0.24	**1.16**	*n.s*
122	20	19	**0.94**	0.27	**1.02**	0.23	**1.09**	*n.s*
142	14	18	**0.95**	0.21	**1.02**	0.24	**1.07**	*n.s*
164	14	15	**0.92**	0.28	**1.02**	0.22	**1.11**	*n.s*
190	11	8	**1.00**	0.19	**1.01**	0.19	**1.01**	*n.s*
220	10	14	**0.88**	0.28	**1.09**	0.23	**1.24**	*0.056*
255	7	7	**0.86**	0.21	**1.06**	0.29	**1.23**	*n.s*
295	11	10	**0.89**	0.28	**0.96**	0.19	**1.07**	*n.s*
342	9	14	**0.93**	0.32	**1.07**	0.23	**1.15**	*n.s*
396	11	12	**0.77**	0.26	**1.01**	0.15	**1.32**	*0.010*
459	13	14	**0.99**	0.19	**1.05**	0.25	**1.05**	*n.s*
531	9	17	**0.90**	0.14	**1.02**	0.19	**1.13**	*n.s*
615	12	17	**0.96**	0.18	**1.04**	0.19	**1.09**	*n.s*
712	12	20	**0.84**	0.15	**1.03**	0.23	**1.24**	*0.013*
825	17	16	**0.80**	0.23	**1.06**	0.25	**1.33**	*0.004*
955	15	14	**0.91**	0.21	**1.03**	0.22	**1.14**	*n.s*
1106	17	13	**0.92**	0.25	**1.08**	0.27	**1.18**	*n.s*
1281	12	15	**0.92**	0.20	**1.06**	0.25	**1.14**	*n.s*
1484	11	10	**0.94**	0.17	**0.97**	0.18	**1.03**	*n.s*
1718	7	13	**1.06**	0.19	**1.07**	0.28	**1.00**	*n.s*
1990	9	7	**0.93**	0.26	**1.09**	0.29	**1.18**	*n.s*
2305	5	3	**1.08**	0.20	**1.04**	0.19	**0.97**	*n.s*
2669	3	3	**1.23**	0.10	**0.96**	0.24	**0.79**	*n.s*
3091	1	1	**1.12**		**1.24**			
3580	4	2	**1.11**	0.20	**1.06**			
4145	3	2	**0.91**	0.18	**0.91**			
4801	3	0	**0.88**	0.41				
max							1.36	

Table S7c – DLS analysis – Numeric data presented in Figure 6c_IgM

rh (nm)	Ctrl (n)	RA (n)	Ctrl IgM mean	Ctrl IgM SD	RA IgM mean	RA IgM SD	IgM RA/Ctrl	*p*
1.7	1	0	**1.28**					
2.7	0	1			**0.79**			
3.6	0	4			**0.97**	0.26		
4.2	1	1	**0.04**		**0.85**			
4.8	1	1	**0.49**		**0.53**			
5.6	3	4	**0.53**	0.42	**0.91**	0.29	**1.71**	*n.s*
6.5	4	7	**0.61**	0.38	**0.83**	0.42	**1.36**	*n.s*
7.5	8	9	**0.54**	0.34	**0.85**	0.43	**1.60**	*n.s*
8.7	13	14	**0.90**	0.46	**0.93**	0.33	**1.03**	*n.s*
10.1	12	14	**0.90**	0.41	**1.03**	0.43	**1.15**	*n.s*
11.7	12	12	**0.84**	0.25	**1.05**	0.48	**1.25**	*n.s*
13.5	16	17	**0.80**	0.36	**0.93**	0.41	**1.16**	*n.s*
15.7	19	15	**0.96**	0.37	**0.98**	0.43	**1.03**	*n.s*
18.2	15	16	**0.73**	0.35	**0.96**	0.39	**1.32**	*n.s*
21.0	17	16	**0.87**	0.33	**1.02**	0.39	**1.17**	*n.s*
24.4	20	18	**0.82**	0.39	**1.04**	0.45	**1.27**	*n.s*
28.2	10	20	**0.83**	0.42	**1.04**	0.41	**1.26**	*n.s*
32.7	18	20	**0.80**	0.37	**1.11**	0.40	**1.38**	*0.020*
37.9	16	15	**0.87**	0.40	**0.97**	0.43	**1.12**	*n.s*
43.8	22	16	**0.81**	0.41	**1.11**	0.38	**1.38**	*0.024*
50.8	17	14	**0.78**	0.43	**1.00**	0.39	**1.28**	*n.s*
58.8	15	16	**0.83**	0.43	**0.99**	0.42	**1.19**	*n.s*
68.0	18	16	**0.82**	0.37	**0.93**	0.37	**1.14**	*n.s*
78.8	24	18	**0.85**	0.38	**0.86**	0.43	**1.01**	*n.s*
91.2	18	13	**0.75**	0.35	**1.02**	0.46	**1.36**	*n.s*
106	12	18	**0.87**	0.32	**0.95**	0.41	**1.09**	*n.s*
122	20	19	**0.91**	0.42	**0.91**	0.41	**1.00**	*n.s*
142	14	18	**0.83**	0.34	**1.02**	0.43	**1.23**	*n.s*
164	14	15	**0.77**	0.47	**0.98**	0.40	**1.27**	*n.s*
190	11	8	**0.97**	0.38	**0.93**	0.35	**0.96**	*n.s*
220	10	14	**0.75**	0.41	**1.10**	0.35	**1.47**	*0.032*
255	7	7	**0.70**	0.44	**1.00**	0.38	**1.42**	*n.s*
295	11	10	**0.70**	0.37	**0.83**	0.31	**1.19**	*n.s*
342	9	14	**0.89**	0.46	**1.07**	0.33	**1.21**	*n.s*
396	11	12	**0.54**	0.33	**0.83**	0.30	**1.54**	*0.040*
459	13	14	**0.97**	0.34	**1.04**	0.46	**1.07**	*n.s*
531	9	17	**0.74**	0.19	**1.03**	0.40	**1.39**	*0.025*
615	12	17	**0.86**	0.27	**1.04**	0.38	**1.22**	*n.s*
712	12	20	**0.74**	0.30	**1.00**	0.40	**1.34**	*n.s*
825	17	16	**0.72**	0.38	**0.95**	0.45	**1.31**	*n.s*
955	15	14	**0.86**	0.37	**1.01**	0.42	**1.17**	*n.s*
1106	17	13	**0.86**	0.34	**1.12**	0.42	**1.29**	*n.s*
1281	12	15	**0.76**	0.26	**1.05**	0.44	**1.38**	*0.054*
1484	11	10	**0.90**	0.32	**1.00**	0.37	**1.11**	*n.s*
1718	7	13	**1.10**	0.34	**1.17**	0.42	**1.06**	*n.s*
1990	9	7	**0.76**	0.39	**1.04**	0.40	**1.37**	*n.s*
2305	5	3	**1.08**	0.46	**1.31**	0.11	**1.21**	*n.s*
2669	3	3	**1.18**	0.48	**1.17**	0.18	**0.91**	*n.s*
3091	1	1	**1.46**		**1.33**			
3580	4	2	**0.89**	0.28	**0.94**			
4145	3	2	**0.58**	0.21	**1.07**			
4801	3	0	**0.69**	0.46				

Table S8. Distribution of CIC particles in CIC of RA ACPA+RF+ and ACPA-RF- RA patients.

rh	ACPA+RF+ (n=20)		ACPA+RF+ (n=6)		*p*
(nm)	with	w/o	with	w/o	
1.7					
2.7	1	19	0	6	*n.s.*
3.6	3	17	0	6	*n.s.*
4.2	1	19	0	6	*n.s.*
4.8	0	20	1	5	*n.s.*
5.6	2	18	1	5	*n.s.*
6.5	5	15	1	5	*n.s.*
7.5	7	13	2	4	*n.s.*
8.7	10	10	3	3	*n.s.*
10.1	10	10	3	3	*n.s.*
11.7	7	13	4	2	*n.s.*
13.5	11	9	3	3	*n.s.*
15.7	9	11	4	2	*n.s.*
18.2	10	10	2	4	*n.s.*
21.0	13	7	4	2	*n.s.*
24.4	13	7	4	2	*n.s.*
28.2	12	8	4	2	*n.s.*
**32.7**	14	6	1	5	*0.054*
37.9	10	10	3	3	*n.s.*
43.8	11	9	2	4	*n.s.*
50.8	10	10	2	4	*n.s.*
58.8	10	10	2	4	*n.s.*
68.0	11	9	4	2	*n.s.*
78.8	10	10	3	3	*n.s.*
91.2	9	11	4	2	*n.s.*
106	11	9	5	1	*n.s.*
122	11	9	2	4	*n.s.*

142	14	6	2	4	*n.s.*
164	10	10	2	4	*n.s.*
190	4	16	2	4	*n.s.*
220	9	11	1	5	*n.s.*
255	4	16	4	2	*n.s.*
295	4	16	3	3	*n.s.*
342	10	10	4	2	*n.s.*
**396**	7	13	5	1	*0.065*
459	7	13	3	3	*n.s.*
531	11	9	4	2	*n.s.*
615	11	9	5	1	*n.s.*
712	13	7	5	1	*n.s.*
825	10	10	4	2	*n.s.*
955	9	11	1	5	*n.s.*
1106	11	9	3	3	*n.s.*
**1281**	12	8	0	6	*0.017*
1484	10	10	1	5	*n.s.*
**1718**	10	10	0	6	*0.053*
1990	6	14	0	6	*n.s.*
2305	3	17	0	6	*n.s.*
2669	2	18	0	6	*n.s.*
3091	1	19	0	6	*n.s.*
3580	1	19	1	5	*n.s.*
4145	1	19	0	6	*n.s.*
4801					
